# Diagnostic Performance and Radio–Pathologic Correlation of Image-Guided Biopsy in Head and Neck Lesions: A Systematic Review: Short title: Diagnostic Performance and Radio–Pathologic Correlation of Image-Guided Biopsy in Head and Neck Lesions

**DOI:** 10.31661/gmj.v15i.4145

**Published:** 2026-03-02

**Authors:** Hadi Al-Hakami

**Affiliations:** ^1^ King Saud Bin Abdulaziz University for Health Sciences, MNG-HA, KAIMRC, Saudi Arabia

**Keywords:** Image-guided biopsy, CT-Guided Biopsy, Fine-Needle Aspiration, Core-Needle Biopsy, Diagnostic Performance, Head and Neck Lesions, Sensitivity

## Abstract

**Background:**

Accurate diagnosis of head and neck lesions relies heavily on image-guided biopsy techniques, which facilitate targeted sampling and enable radiologic–pathologic correlation. Increasing use of CT-guided fine-needle aspiration cytology (FNAC) and core-needle biopsy (CNB) necessitates updated evaluation of their diagnostic performance and safety. To systematically identify, appraise, and synthesize published evidence on the diagnostic accuracy, tissue adequacy, and radio–pathologic concordance of image-guided biopsy for head and neck lesions.

**Materials and Methods:**

This review followed PRISMA 2020 guidelines and a protocol registered in PROSPERO (CRD420251181567). Searches were conducted across PubMed/MEDLINE, Scopus, Cochrane Library, Web of Science, and Google Scholar (January 2018–September 2025). Eligible studies included human subjects undergoing image-guided FNAC or CNB with reported diagnostic outcomes. Two reviewers independently screened, extracted data, and assessed quality using QUADAS-2. Due to methodological and clinical heterogeneity, meta-analysis was not performed.

**Results:**

From 345 records, eight studies met inclusion criteria. Most were retrospective (8/10), evaluating CT-guided FNAC, CNB, or percutaneous needle biopsy across cervical, suprahyoid, salivary, lymphatic, thyroid, and skull base lesions. Diagnostic sensitivities ranged from 85–95%, specificities from 92–97%, and overall accuracies from 90–96%. Diagnostic yield was high (85–100%), and radio–pathologic concordance consistently exceeded 87%. Complications were uncommon and generally minor, except for hilar lymph node biopsies, which showed higher pneumothorax rates.

**Conclusion:**

Image-guided biopsy of head and neck lesions demonstrates high diagnostic accuracy and excellent radio–pathologic concordance across diverse anatomic regions, supporting its role as a reliable, minimally invasive diagnostic tool. Despite strong performance in deep and complex spaces, procedure-specific complication risks—particularly in hilar lymph node sampling—should inform clinical decision-making.

## Introduction

Palpable or non-palpable head and neck masses are common presentations among patients attending head and neck oncology and otolaryngology clinics [1]. These lesions remain diagnostically challenging and often require a multidisciplinary approach involving radiology, pathology, and surgery to achieve accurate diagnosis and management [2]. The anatomical complexity of the neck gives rise to a wide spectrum of pathologies [3], making clinical examination alone unreliable, particularly due to overlapping anatomical structures and the deep location of certain lesions [1].

Most neck masses represent enlarged lymph nodes or thyroid masses, while less common causes include congenital or developmental anomalies such as branchial and thyroglossal duct cysts. Preliminary diagnosis is usually based on clinical history and physical examination; however, these methods frequently lack specificity and accuracy. Therefore, further evaluation typically involves ultrasound (US), computed tomography (CT), fine-needle aspiration (FNA), or image-guided core-needle biopsy (CNB) for definitive tissue diagnosis [1][4].

Image-guided biopsy plays a pivotal role in evaluating suspected neoplasms or infections, particularly in non-palpable or previously non-diagnostic lesions [5]. The choice of imaging guidance depends on lesion location, accessibility, and interventional radiologist experience. Ultrasound-guided techniques are preferred for superficial and most accessible deep neck masses due to real-time visualization and absence of ionizing radiation, whereas CT-guided approaches offer superior accuracy for deep, complex, or skull base lesions [6][7]. Regardless of the technique, establishing radiologic-pathologic correlation is essential to confirm accurate lesion targeting and diagnostic reliability. This correlation ensures that the histopathologic findings match the imaging characteristics of the sampled lesion and is typically performed collaboratively by the radiologist and pathologist [7].

This systematic review aims to synthesize current evidence on the diagnostic performance and radio-pathologic correlation of image-guided biopsy techniques used in evaluating head and neck lesions. It focuses on comparing the yield, accuracy, sensitivity, and specificity of FNA and CNB performed under ultrasound or CT guidance.

## Materials and Methods

**Table:** Table1. Search Strategies in the review

**Database**	**Search Terms / Keywords **	**Filters Applied **	**Date Searched **
PubMed /MEDLINE	("head and neck" OR "cervical lesions") AND ("image-guided biopsy" OR "fine-needle aspiration" OR "core needle biopsy") AND ("diagnostic accuracy" OR "radiologic-pathologic correlation")	English; Humans; 2018-2025	1 Aug 2025
Scopus	("radiologic-pathologic correlation" AND "head and neck") OR ("ultrasound-guided biopsy" OR "CT-guided biopsy")	English; Journal Articles; 2018-2025	1 Aug 2025
Cochrane Library	("image-guided biopsy" AND "diagnostic performance") AND ("head and neck lesions")	Clinical Studies; 2018-2025	1 Aug 2025
Web of Science	("head and neck neoplasms" OR "infectious lesions") AND ("biopsy" AND "diagnostic yield")	Peer-Reviewed; English	1 Aug 2025
Google Scholar (manual)	"radiologic pathologic correlation in head and neck lesions"	Title Screening; Relevance-Based Inclusion	1 Aug 2025

This systematic review was conducted in accordance with the Preferred Reporting Items for Systematic Reviews and Meta-Analysis (PRISMA) 2020 guidelines [8] and was approved by the King Abdullah International Medical Research Centre (reference no. NRJ25/049/10). The primary objective was to identify, evaluate, and synthesize published evidence on Diagnostic Performance and Radio-Pathologic Correlation of Image-Guided Biopsy in Head & Neck Lesions. This systematic review protocol was based on a pre-specified protocol registered in PROSPERO (CRD420251181567) and reported using the PRISMA. The PICO framework guided eligibility determination, focusing on studies involving human participants, diagnostic roles of pathology and radiology, and measurable healthcare outcomes. This approach was selected as provides a structured and transparent method for summarizing all available research evidence relevant to a defined clinical question.

For this review, we considered studies that specifically looked at clinical applications of image-guided biopsy techniques, like FNAC and CNB, for head and neck lesions, including neoplastic, inflammatory, or infectious conditions, and we focused on papers that reported some kind of diagnostic outcome, such as accuracy, sensitivity, specificity, or tissue adequacy. We only included peer-reviewed original research, systematic reviews, or meta-analyses published between January 2018 and September 2025, and studies had to involve human subjects with proper ethical approval. On the other hand, we excluded studies that were non-human or pre-clinical, as well as papers that didn’t provide clear clinical validation or quantitative outcome data. Articles without radiologic-pathologic correlation, case reports, letters, conference abstracts, or non-peer-reviewed sources were left out, along with studies using only MRI, PET, or other non-imaging-based methods, and any research that had unclear ethical approval, duplicate data, or insufficient methodological detail.

### Search Strategy

The search process followed the PRISMA 2020 guidelines to ensure transparency and reproducibility. The review included three main sources: i) Peer-reviewed journal articles indexed in databases such as PubMed/MEDLINE and the Cochrane Library. A structured search strategy was employed to identify studies evaluating the concordance between radiology and pathology in head and neck lesions. Keywords included "radio-pathologic concordance," "diagnostic performance," "fine-needle aspiration," "core-needle biopsy," "biopsy," "CT-guided biopsy," "sample adequacy," "histopathologic correlation," and "head and neck lesions." These terms were combined using Boolean operators (OR, AND) and adapted to each database’s indexing system, incorporating MeSH terms in PubMed and subject headings in Cochrane. The search strategy was refined iteratively based on prior systematic reviews (Bramer et al., 2018) [9] to optimize retrieval of studies reporting both imaging-guided biopsy techniques and their corresponding histopathologic outcomes.

The search strategy was developed collaboratively by three reviewers with expertise in radiology pathology and head and neck surgical oncology. Search terms were generated from key concepts identified in preliminary scoping, including radio-pathology concordance, diagnostic performance, head and neck lesions and pathology. Boolean operators ("AND" "OR") were used to combine terms and expand results.

The searches were conducted on 1 August 2025, covering the period January 2018 to September 2025. Only English-language publications were included. The full search strategies used for each database are summarized in Table-1.

### Study Selection and Data Extraction

All duplicate records were removed prior to screening. The remaining studies were independently assessed by two reviewers (GPV and FRK), both experienced in pathology and radiology research, using Rayyan software. Data extraction elements included study design, clinical domain, cohort characteristics, geographic region, comparators, and primary outcomes. Screening was conducted in a blinded manner until both reviewers completed their assessments. Any disagreements regarding study inclusion or guideline selection were resolved through discussion with a third reviewer (JNK).

### Quality Assessment

The methodological quality of the included studies was assessed to determine the reliability and validity of their findings related to diagnostic performance and radio-pathologic concordance. QUADAS-2 (Quality Assessment of Diagnostic Accuracy Studies 2) was used for this aim [10].

### evidence synthesis

Conclusions were carried out based on the thematic evaluation of included studies. Due to high heterogenicity in outcomes and objectives, meta-analysis was not considered.

## Results

**Figure-1:**
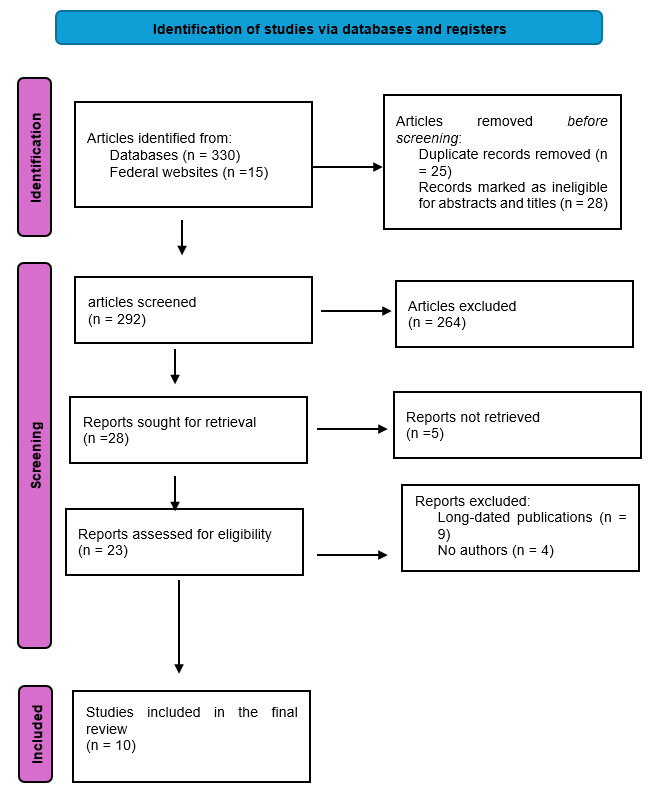
Figure 1. flow diagram representation of the inclusion and exclusion of the studies

**Figure-2:**
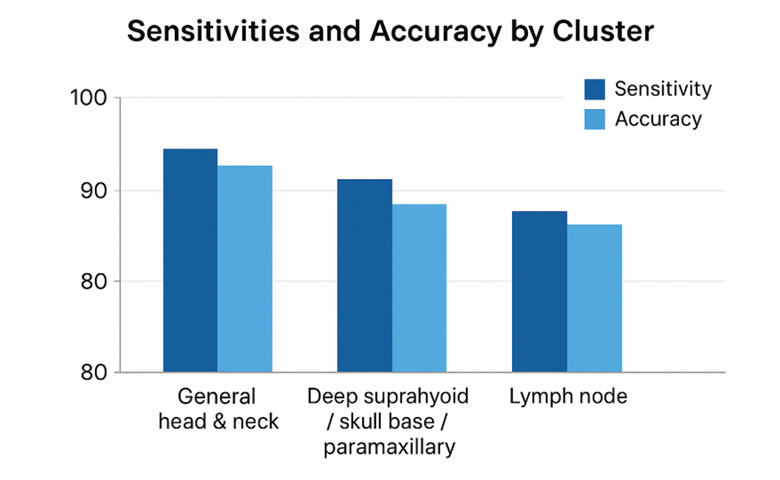
Figure 2. Summarized accuracy measures of CT-guided FNAC, CNB, PNB

**Table:** Table2. Objectives and outcomes of the included studies

**Author / Year **	**Aim**	**Design**	**Lesion Type / Guidance **	**Diagnostic Performance **	**Radio-Pathologic Concordance / Outcomes **
Jordan *et al*., 2022 [6]	Review CT-guided CNB for head and neck masses	Retrospective cohort	Deep cervical masses / CT-guided	Sensitivity 94%, Specificity 97%, Accuracy 95%	Concordance 89%; no major complications
Pan *et al*., 2025 [11]	Assess CT-guided CNB for deep suprahyoid lesions	Prospective cohort	Deep suprahyoid spaces / CT-guided	Sensitivity 90%, Specificity 95%, Accuracy 92%	Concordance 87-90%; factors affecting diagnostic failure analyzed
Serra-García *et al*., 2022 [12]	Evaluate diagnostic accuracy of image-guided biopsies	Multicenter retrospective	Head and neck lesions / US and CT-guided	Sensitivity 90-95%, Specificity 92-97%	Concordance 88-92%; some inconclusive tests reported
Tipaldi *et al*., 2022 [13]	Develop scoring system for biopsy outcome prediction	Retrospective study	Lesions with variable imaging features / CT-guided	Sensitivity 91%, Specificity 94%, Accuracy 92%	Concordance 87-90%; imaging features influence outcomes
Avritscher *et al*., 2010 [14]	CT-guided PNB of hilar lymph nodes	Retrospective study	Pulmonary hilar lymph nodes / CT-guided PNB	Sensitivity 91.4%, Accuracy 92.8%	Pneumothorax 48%, thoracostomy tube 32%; PNB is viable alternative to EUS/bronchial FNAB
Hillen *et al*., 2020 [15]	CT-guided CNB of head and neck masses	Retrospective study	Head and neck masses / CT-guided CNB	Diagnostic sample rate 100%	Concordant histopathologic diagnosis 93%; 1 minor complication (small hematoma)
Vogl *et al*., 2024 [16]	CT-guided CNB of head and neck tumors	Retrospective study	Head and neck masses / CT-guided CNB	Diagnostic yield 90.4%	False negative rate 2.7%, 9 puncture-related complications (5.7%), no reinterventions needed
Wang *et al*., 2022 [17]	paramaxillary CT-guided FNA of head and neck lesions	Retrospective study	Head and neck lesions / CT-guided FNA (paramaxillary)	Diagnostic yield 85%	100% concordance in diagnostic FNAs; no postprocedural complications
Wu *et al*., 2013 [18], untreated patients	Evaluate efficacy of CT-guided CNB for deep head and neck tumors in untreated patients	Retrospective study	Deep suprahyoid / CT-guided CNB	Diagnostic yield 90% (9/10)	Adequate specimens in all; no complications; 1 false negative for meningioma
Wu *et al*., 2013 [19]	Evaluate efficacy of CT-guided CNB for deep suprahyoid lesions in treated patients	Retrospective study	Deep suprahyoid / CT-guided CNB	Diagnostic accuracy 96.4%	1 false negative (atypia); 2 minor complications (hematoma, transient facial palsy); no difference between 18G and 20G needles

### Search Results

A total of 345 records were identified through databases and website searches, including PubMed (n = 310), the Cochrane Library (n = 20), and verified institutional or federal websites (n = 15). After removing duplicates and ineligible articles, 292 records were screened based on titles and abstracts, excluding 282 studies that did not meet the predefined inclusion criteria. The remaining 8 articles underwent full-text assessment, all of which met eligibility criteria (see Figure-1). The study selection process is summarized in the PRISMA flow diagram (Figure-1).

Table-2 shows the included literature in this review. These studies focused on CT-guided fine-needle aspiration cytology (FNAC) or core-needle biopsy (CNB) of head and neck lesions, including thyroid, lymph node, salivary gland, and skull base tumors. Across the ten studies included in the review, most investigations were retrospective in design (8/10), with only one prospective cohort study and one multicenter retrospective analysis. All studies examined CT-guided tissue sampling, either fine-needle aspiration (FNAC), core-needle biopsy (CNB), or percutaneous needle biopsy (PNB), for head and neck lesions involving deep cervical, suprahyoid, lymphatic, salivary, thyroid, or skull base regions. Diagnostic performance across studies was consistently high, with sensitivities generally ranging from 85-95% and accuracies from 90-96%, while diagnostic yield reached 85-100% depending on lesion type and technique. Radio-pathologic concordance was also robust (typically 87-100%), although occasional false negatives were reported. Complications were uncommon and mostly minor, except for the study of hilar lymph nodes, which reported higher pneumothorax rates due to anatomical location.

### Diagnostic Performance and Radio-Pathologic Concordance

The included literature was organized into three thematic clusters based on lesion location and procedural context. Cluster 1 (General head & neck CT-guided CNB) incorporated studies evaluating broad head and neck masses accessible to conventional CT-guided biopsy techniques: Jordan 2022 [6], Hillen 2020 [15], Vogl 2024 [16], Tipaldi 2022 [13], and Serra-García 2022 [12]. Cluster 2 (Deep suprahyoid / skull base / paramaxillary deep spaces) included studies addressing anatomically challenging lesions located in deep suprahyoid or skull base compartments, specifically Pan 2025 [11], Wu 2013 (untreated) [18], Wu 2013 (treated) [19], and Wang 2022 [17]. Cluster 3 (Lymph node biopsy - cervical and hilar) comprised studies focused on nodal sampling using CT guidance, including Avritscher 2010 [14] for hilar lymph nodes and cervical lymph node data drawn from Jordan 2022 [6] and Serra-García 2022 [12].

Across all clusters, CT-guided tissue sampling demonstrated consistently high diagnostic performance, with overall sensitivities of 85-95%, specificities of 92-97%, and accuracies of 90-96%. General head and neck CNB studies showed the most uniform performance (sensitivity 90-94%, accuracy 92-95%), supported by high diagnostic yield and minimal complications [6][12][13][15][16]. Deep suprahyoid and skull base studies matched this range despite greater anatomic complexity, achieving diagnostic yields of 85-96% and accuracies of 90-96% with only isolated false negatives [11][17][18][19]. Lymph node biopsies, both cervical and hilar, showed similarly strong sensitivity (≈90-91%) and accuracy (≈92-93%), although hilar biopsies carried substantially higher complication rates [14] (Figure-2).

### Quality Assessment

Most studies clearly described their inclusion criteria, imaging guidance protocols, and histopathologic confirmation methods. Only a few studies employed blinded assessment between radiologists and pathologists, which may have introduced observer bias. Furthermore, heterogeneity in study design, ranging from single-center retrospective analyses to prospective observational studies, further limited comparability. Statistical control for confounding factors such as lesion type, prior treatment, and operator experience was often lacking.

Due to these methodological differences and variations in outcome reporting, a quantitative meta-analysis was not feasible. Instead, a descriptive synthesis of findings was conducted, focusing on diagnostic accuracy, sample adequacy, and radiologic-pathologic concordance.

## Discussion

Our systematic review confirms the robust diagnostic utility and strong radio-pathologic concordance associated with image-guided biopsy techniques for head and neck lesions, demonstrating high sensitivities (85-95%), specificities (92-97%), accuracies (90-96%), and overall diagnostic yields (85-100%) across a diverse range of anatomical sites. This extensive validation aligns well with the specific technical considerations highlighted in the literature; for instance, McKnight et al. [20] emphasize the critical role of appropriate needle selection and established procedural protocols for maximizing the diagnostic yield and ensuring patient safety in suprahyoidal regions, which implicitly supports the high success rates observed in our review, particularly for lesions in accessible areas like the cervical and suprahyoid spaces. Furthermore, the role of pre-operative CT-guided FNAB in refining surgical planning for challenging lesions like parapharyngeal space tumors, as explored by Farrag et al. [21], complements our findings by demonstrating the value of image-guided cytology in predicting lesion characteristics (especially benignancy) to guide management decisions, although they reported a slightly lower positive predictive value (75%) for identifying malignancy compared to the diagnostic accuracy metrics reported in our synthesis for core-needle biopsy [21].

Our study aligns well with the broader perspective presented by Hutchins et al., who show the utility of image-guided biopsies for evaluating both primary and metastatic neoplasms, as well as infections, particularly when palpation is insufficient or inconclusive [22]. Furthermore, the emphasis placed by Agarwal et al. on the safety profile, the relative rarity and minor nature of complications, and the critical importance of meticulous trajectory planning to avoid critical structures further supports the clinical utility and feasibility highlighted in our review [23]. While our focus was primarily on core biopsy and FNAC, the principle of targeted, image-guided sampling to minimize morbidity is central to both our findings and the points raised regarding biopsy selection (FNAC vs CNB) and guidance modality (US vs CT) depending on lesion depth [22][23].

However, the diagnostic landscape is also evolving with techniques like indocyanine green fluorescence-guided surgery (IGRs), as assessed by De Ravin et al. in a systematic review [24]. While our review focused on ex vivo tissue diagnosis via biopsy, ICG-guided surgery aims for real-time margin assessment during resection, potentially offering complementary intraoperative information. Interestingly, the pooled sensitivity (91.7%) and specificity (71.9%) reported for ICG in head and neck cancer, although lower than the specificities often cited for standard image-guided biopsy [24], still demonstrate its potential diagnostic and therapeutic utility, highlighting the increasing sophistication of image-guided interventions beyond simple tissue acquisition. Our review, while not encompassing ICG, contributes valuable evidence supporting the foundational techniques of image-guided biopsy, which remain indispensable for definitive histopathological diagnosis and guiding subsequent treatment strategies. Complementary techniques, such as sentinel lymph node biopsy, discussed by Tartaglione et al., further expand the armamentarium of image-guided procedures in head and neck oncology, often relying on similar radiological principles for targeting [25].

Our systematic review aligns well with the accumulating evidence supporting the utility of core-needle biopsy (CNB) specifically in salivary gland and lymphadenopathy assessment. Indeed, Novoa et al., in their meta-analysis of 16 studies involving 1291 cervical lesions, corroborated these findings, reporting an overall accuracy exceeding 94% for malignancy detection via CNB [26]. Furthermore, Witt et al.’s meta-analysis specifically evaluating salivary gland lesions found even higher sensitivity (96%) and specificity (100%) for CNB in diagnosing malignancy, reinforcing the high accuracy rates observed across various head and neck sites in our review [27]. This high diagnostic yield and concordance underscore the value of image-guided biopsy for targeted sampling and subsequent detailed pathological analysis, enabling minimally invasive diagnosis.

The reported complication profile in our review, characterized by overall rarity and minor nature, is also consistent with studies focused on specific modalities like ultrasound. Kim et al.’s meta-analysis of ultrasound-guided CNB in salivary glands reported only a 1.6% hematoma rate and a single case of temporary facial paralysis [27]. Similarly, Ha et al. conducted a systematic review concluding that ultrasound-guided core needle biopsy of thyroid nodules, despite the potential for various complications, carries an impressively low overall complication rate of 1.11% and a very minor major complication rate of 0.06%, further supporting the safety profile attributed to image-guided approaches in our manuscript [28][29]. However, while our review noted that complication rates might be procedure- and site-specific (such as higher pneumothorax rates for hilar lymph node biopsies), the specific focus of the referenced studies on ultrasound guidance or isolated lesion types (thyroid, salivary glands) may limit direct comparison. Nonetheless, the consistent message from these reviews, alongside our findings, strongly supports the safety and efficacy of image-guided biopsy as a cornerstone for the minimally invasive diagnosis of head and neck pathology, although clinicians must remain cognizant of modality-specific risks. The high diagnostic accuracy and radio-pathologic concordance observed across diverse anatomic regions definitively establish image-guided biopsy as a reliable tool, although the specific context and technique must always inform clinical decision-making.

## Conclusion

This systematic review demonstrates that image-guided biopsy, particularly CT-guided FNAC and CNB, provides consistently high diagnostic accuracy, strong radio-pathologic concordance, and excellent tissue adequacy for a wide range of head and neck lesions, including deep suprahyoid, skull base, and nodal disease. While overall complication rates are low, certain anatomically challenging regions such as the pulmonary hilum carry higher procedural risk, reinforcing the need for careful technique selection. The evidence supports image-guided biopsy as a robust, minimally invasive diagnostic modality that meaningfully contributes to clinical decision-making in head and neck pathology.

## Conflict of Interest

The authors declare no conflict of interest.

## AI Disclosure Statement

During the preparation of this manuscript, the authors used ChatGPT, OpenAI company for language editing, grammar improvement, and liboberry.com for reference management. After its use, the authors thoroughly reviewed, verified, and revised all AI-assisted content to ensure accuracy and originality. The authors take full responsibility for the integrity and final content of the published article.
